# Critical issues facing the animal and food industry: a Delphi analysis

**DOI:** 10.1093/tas/txaa213

**Published:** 2020-11-19

**Authors:** Kevan W Lamm, Nekeisha L Randall, Francis L Fluharty

**Affiliations:** 1 Department of Agricultural Leadership, Education and Communication, University of Georgia, Athens, GA; 2 Department of Learning, Leadership, and Organization Development, University of Georgia, Athens, GA; 3 Department of Animal and Dairy Science, University of Georgia, Athens, GA

**Keywords:** dairy industry, food security, meat production, population

## Abstract

By the year 2050, the world's population is estimated to increase by approximately 2.1 billion people. For the sake of food security and safety, it is vital for the animal and food industry to act now in preparation for future consumption needs. The study at hand explored the most critical issues facing the field, according to industry experts. Using Delphi and constant comparative methods, seven thematic categories emerged that serve as overarching areas for attention: Industry Image and Relationship with the Public, Workforce Development and Pipeline, Economic and Environmental Sustainability, Animal and Human Health/Well-Being, Production and Distribution Efficiency, Government Regulations and Relationship with Legislative Leaders, and Relationship with Higher Education/Researchers. Findings and recommendations on how the industry can move forward in light of future challenges and opportunities are explored.

## INTRODUCTION

The National Research Council of the National Academies (2015) has called for the food animal industry to prepare for the larger food consumption demands that come with a growing global population. Currently, there are more than 7.6 billion people in the world (United States Census Bureau, 2020); many of whom rely on animal protein provided by animal agriculture. By 2050, the United Nations (2019) foresees the current world population increasing to 9.7 billion people. “Sustainably meeting the nutritional needs of this population and its demand for animal products will require a significant research and development (R&D) investment so that the productivity of today can be sufficiently enhanced to meet the much heightened demands” of tomorrow (National Research Council of the National Academies, 2015, p. vii). In 2017, the per capita supply of red meat, poultry, and fish totaled 143.9 pounds in the United States, each with comparative percentages of 51%, 42%, and 7%, respectively (Bentley, 2019). Also, in the United States alone, the per capita consumption of dairy products totaled approximately 646 pounds in 2018 (United States Department of Agriculture, 2020). It is projected that these needs, met by the food animal industry, will increase over the next 30 years.

According to Hernandez-Castellano et al. (2019), the majority of the world's population will increase in developing countries such as those located in the tropical areas of Africa, Asia, Latin America, and the Caribbean, aligning with current trends involving increased milk and meat production in developing, rather than developed, countries (Thornton, 2010; Guyomard et al., 2013). As such, future demand for animal products may undoubtedly influence trade dependence and food prices (Guyomard et al., 2013). In addition to sustainable practices and science and technology advancements (Thornton, 2010), enhanced animal agricultural research is critical to food security as more opportunities and challenges related to the land, water, and energy needed for production arise and the risk for disease and trade issues increase with globalization (Thornton, 2010; National Research Council of the National Academies, 2015). Such issues may relate to environmental concerns as a result of animal product production (Guyomard et al., 2013; Hernandez-Castellano et al., 2019). Additionally, observations of increased animal product consumption in developing countries that are seeing more urbanization are top of mind for agricultural forecasters (National Research Council of the National Academies, 2015). In urbanized areas in the United States and abroad, “[c]ompared with the less diversified diets of the rural communities, city dwellers have a varied diet rich in animal proteins and fats…characterized by higher consumption of meat, poultry, milk and other dairy products” (World Health Organization, n.d.). More urbanization may also influence how the next generation perceives the societal importance of livestock, animal welfare, and how concepts, such as organic farming and food labeling, affect health perceptions (Bobeck et al., 2014). Moreover, to attract urban youth and young adults to the industry to prepare the next generation of farmers, ranchers, and leaders, the conversation about how to evolve current animal and dairy science academic departments and make the related curriculum broader in scope (Bobeck et al., 2014; Sterle and Tyler, 2016) must continue. Thus, issues related to how the industry prepares for the future extend from farms to classrooms.

Related to the parallel of urbanization and development, income is positively correlated to animal protein consumption. In fact, the more income a population has, the more eggs, milk, and meat are consumed, even compromising the value of staple foods (World Health Organization, n.d.). Consumption of animal-related products is not only related to consumers' taste preferences, but also their health. According to the World Health Organization (n.d.), the high-value protein, micronutrients, and vitamins provided by such products are desired by many consumers, especially those in developing countries. It is important to note, however, that changing socio-cultural values affect consumer preferences and opinions about animal welfare, environmental consequences, and healthy food options, which in turn influence production demands and consumption trends (World Health Organization, n.d.; Thornton, 2010; National Research Council of the National Academies, 2015; Karavolias et al., 2018). In relation to the importance of communication with consumers and the general public, Capper and Yancey (2015) argue that how the animal science community prepares itself for consumer and media opinions (which inevitably affect policy and practices) will positively or negatively impact the sustainability of the industry itself.

It would be challenging to take the aforementioned matters into holistic consideration without also bearing in mind unexpected situations such as the COVID-19 global pandemic, a drastic situation that has long-reaching economic losses and impacts on farmers, ranchers, distribution standards, consumers, and societies (Greene et al., 2020). It will also be important to monitor local food markets, their impact on local economies (Rossi et al., 2017), and how micro-level changes in those economies possibly affect the industry on a macro-scale.

Though there is still uncertainty revolving around how the food animal community will address foreseen and unforeseen challenges in the future (Thornton, 2010), acknowledging the state of the industry now and using current data to project the future is an intentional and worthwhile effort that is an investment not only for the industry, but for all consumers globally. Given the myriad of factors affecting the industry in the coming decades, the purpose of the study at hand was to pinpoint critical industry issues. This purpose relates to gaining a better understanding of how the goal of ensuring “the availability of high-quality animal protein products” (Hernandez-Castellano et al., 2019, p. 1010) can be achieved now and for generations to come. Such exploration is in alignment with the National Research Council of the National Academies' (2015) call for additional investment in animal and food industry research.

## MATERIALS AND METHODS

The study at hand utilized Dalkey and Helmer's (1963) Delphi method to gather the necessary data for advancing animal industry research and practice. Based on group communication and consensus-building processes (Ludwig, 1997; Terry and Osborne, 2015), the Delphi method involves repeated questioning to research participants who serve as a panel of experts (Dalkey and Helmer, 1963). Such participants are chosen as experts because of how well their knowledge and experience connect to the research topic (Costello and Rutherford, 2019). The Delphi method usually begins with panelists being given an open-ended question (Terry and Osborne, 2015). Panelists are then given subsequent questions or tasks, based on their initial responses, in an effort to generate more precise and agreed-upon information. A unique strength of the Delphi method is the ability it offers researchers to combine quantitative and qualitative methodology to eventually gather data based on expert consensus (Gamon, 1991). Such a method is also beneficial for analyzing the current state of an organization or industry, pinpointing trends, and realizing areas where potential change can be made (Ludwig, 1997).

Due to the Delphi method being suitable when the strength of a study is dependent upon the expertise of the research participants, perspectives from industry leaders and corresponding higher education faculty were garnered for this study. The expert panel consisted of 31 participants, all of whom reflected various parts of the animal and dairy science community. Participants held positions such as: Farm Bureau employees, state fair director, agricultural writer, commissioner of agriculture, food product industry representatives, veterinarians, and county Extension agents. Other panelists also identified as beef, pork, and milk and dairy producers and commodity group representatives. The panel of experts was primarily sourced from a single state in the southeastern United States. To expand the extent of potential responses, the first round of data collection included responses from faculty members from an Animal and Dairy Sciences department housed at a Research I university also located in the southeastern part of the United States. The inclusion of academic respondents in the first round of the process was done to mitigate the potential for response bias, specifically, “if experts are all professionals in the same area, bias toward a professional agenda may emerge” (Garson, 2014, Location No. 462). Therefore, including both academic and professional responses from the first round helped to guard against a limited set of options to consider in subsequent rounds of the process (Garson, 2014).

Panelists participated in three rounds of the Delphi process, which was administered using the on-line Qualtrics Survey Software (Qualtrics, Provo, UT). Following the Tailored Design Method (Dillman et al., 2008), panelists received an electronic message before surveys were distributed to inform them of the study's purpose and process. Within two days, the same group received personalized links, signifying the official start of the research process. A minimum of three messages were also sent out every three to seven days as a friendly reminder to those who had not sent in their responses. A pre-notice, an official invitation to participate in the study, and reminder messages were forms of communication done during the entirety of the Delphi process, which included three rounds.

For the first round, participants received the following prompt: “In your opinion, what are the most critical issues facing the animal and food industry? Please use a word or short description to briefly describe up to five of the top critical issues.” The qualitative responses were analyzed using version 7.0.23 of the Dedoose data analysis software (Dedoose, Manhattan Beach, CA) and were used as the foundational items for the quantitative portion of the Delphi method. Specifically, responses from round one created the items panelists saw on a survey they received in round two. Using the survey and a five-point Likert scale (1 = “Not at all important,” 2 = “Somewhat important,” 3 = “Important,” 4 = “Very important,” and 5 = “Extremely important”), panelists had an opportunity to rank the critical issues shared by fellow panelists in round one based on level of importance. Afterwards, round two responses were analyzed using SPSS version 25.0 (IBM Corporation, Armonk, NY). Survey items with mean scores higher than 3.5 (Garson, 2014) were retained and used in the third, and final, round. Although the purpose of round two was to generate a refined list of critical issues, the purpose of the last round was to generate a final list based on group consensus. To achieve this objective, panelists were given a list of all retained items and were asked to answer “Yes” or “No” to the question of if individual items should be retained once more. Similar to how data was analyzed in round two, SPSS (IBM Corporation, Armonk, NY) was used to analyze subsequent round three data. Survey items with agreement percentages greater than 80% were retained and used to create the final, expert-created list of critical issues. Response rates for rounds one, two, and three were 87% (27 respondents), 84% (26 respondents), and 65% (20 respondents), respectively.

To extrapolate additional meaning from the resulting list of issues generated in round three, the qualitative research concept of the constant comparative method (CCM; Glaser, 1965) informed the final stage of data analysis. As the name suggests, the CCM uses comparison to analyze, code, and make meaning of data. The method is connected to the qualitative methodology of grounded theory, but aspects of it are commonly used for thematic analysis in quantitative research as well as qualitative research not specific to grounded theory. The CCM involves continuous development (Glaser, 1965) and is, therefore, similar to the Delphi process given that it builds upon itself. First, codes are given to data items to describe and label them. Then, data items and corresponding codes are compared to each other to determine what categories are most appropriate to create; categories conceptualize the codes (Glaser and Strauss, 1967). Lastly, the process of comparing categories to other categories generates more information about the abstract meaning of the data, giving the researcher a higher level of conceptualization and theoretical properties with which to work (Glaser, 1965; Glaser and Strauss, 1967). Names of categories can be inspired by research participant language or interpretation generated by the data analyst. For the study at hand, all remaining critical issues were reviewed by hand and the analysis of similarities and differences resulted in color-coded groupings, codes, and subsequent categories. The CCM process took place over a series of days to allow for multiple reviews and better scrutiny of the chosen categorization and conceptualization.

## RESULTS

In round one, the panel provided 110 responses to the critical issues prompt. Those responses were combined to create one list, which panelists used to express views about each issue's level of importance in round two. As a result, “Industry image” and “Consumer perception of food animal industry” emerged as the two critical issues with the most level of importance, both with a tied mean score of 4.56. In contrast, “Climate change” had the lowest mean (*M* = 2.84). After instituting the 3.5 mean cutoff, 19 issues were removed and 91 were retained (Table 1) in preparation for round three. In addition to sharing information about the industry's story and image, other themes underlying the top 10 issues involved quality products, quality personnel, and consumer education and opinion.

**Table 1.** Delphi round two results: level of importance for critical animal and food issues (*n* = 91)

Issue	*M*	*SD*
Industry image	4.56	0.651
Consumer perception of food animal industry	4.56	0.583
Labor availability	4.52	0.586
Telling our story as an industry	4.48	0.770
Recruiting the next generation of agricultural leadership with a work ethic	4.40	0.707
Product quality	4.36	0.638
Misinformed Consumers	4.36	0.810
Consumer education	4.36	0.810
Consumer acceptance of current and future production practices (e.g., food safety, animal welfare, antibiotics, genetics, etc.)	4.36	0.700
Relating to the next generation of consumers	4.32	0.627
Customer confusion	4.28	0.614
Food security	4.28	0.792
Capital intensive (rising input costs)	4.28	0.891
Societal demographic shift toward urban areas and the decrease in population and political clout in rural agricultural areas	4.28	0.891
Poor consumer understanding of agriculture	4.24	0.723
Animal well being	4.24	0.779
Awareness of animal protein's role in a healthy diet	4.24	0.779
Public perception of food animal industry	4.24	0.723
Negative perception of animal agriculture	4.24	0.723
Misinformation on animal production practices	4.24	0.723
Young people returning to food animal operations	4.21	0.833
Training a sufficient number of food animal production students to meet the future needs of the industry.	4.21	0.833
Disconnect between production agriculture and general public	4.20	0.764
Gap between academia and industry	4.16	0.850
Import/Export issues	4.16	0.800
Animal welfare	4.16	0.943
Long-term economic prospects for animal agriculture (economic sustainability)	4.12	0.971
Public relations	4.12	0.781
Misinformation on internet	4.12	0.833
Food safety (e.g., safety of meat products)	4.08	0.881
Workforce preparation	4.08	0.702
Fear based information distributed to consumers	4.08	0.909
Education of consumers on where food comes from	4.08	1.077
Too hard for rural students to get into [University]	4.04	1.022
Profitability of agriculture at the producer level	4.04	0.935
Public perception of animal impact on climate change	4.04	1.098
Public opinion about meat consumption	4.04	0.889
Disease (e.g., control, resistance, transmission, etc.)	4.00	0.577
Growing world population	4.00	0.913
Animal Rights advocates	4.00	0.913
Reproductive efficiency	3.96	0.841
Feed efficiency	3.96	0.790
Low profit margin	3.96	1.060
Government economics (taxes/tariffs)	3.96	0.889
Marketing (of food production generally)	3.96	0.735
Different perception between producers and consumers	3.96	0.735
Global politics anti-meat campaigns (e.g., EAT-Lancet)	3.96	1.136
Qualified graduates	3.96	0.935
Feed costs	3.96	0.806
Anti-animal agriculture activism	3.92	1.038
Access to Finance	3.92	0.997
Personnel wanting to work in production agriculture.	3.92	0.862
Poor legislative influence	3.92	1.038
Insufficient processing capacity	3.92	0.759
Being financially sustainable with additional customer and government regulations	3.92	0.997
Costs of everything needed (fluctuations)	3.92	0.830
Shrinking dairy industry	3.88	1.130
Health and robustness of farm animals	3.88	0.881
Adapting to new technologies	3.88	0.781
Efficiency in farming	3.88	0.741
Lack of specialized people	3.84	0.898
Commodity prices (high to buy, low to sell)	3.84	1.028
Government regulations	3.80	1.291
Increase in non-milk substitutes such as almond and coconut “milk”	3.80	1.190
Foreign unequal competition	3.76	0.970
Need for improved traceability and record keeping	3.76	0.879
Collapse of small and mid-size farming	3.76	1.091
Barriers to entry	3.76	0.926
Environmental loads and sustainability (e.g., protection, stewardship, etc.)	3.76	0.831
Continued increasing demand for improved efficiency	3.75	0.794
Lack of large animal DVM	3.72	1.173
Understanding of global impacts	3.72	0.792
Interactions between diet and human well-being/health (mediated by microbiome)	3.72	0.792
Lack of financial support for applied research to answer food animal systems level questions	3.72	0.737
International trade (e.g., barriers, meeting demands to drive exports, unfair import competition, etc.)	3.72	0.843
Need intern program set up for production agriculture	3.72	1.100
Lawsuits	3.72	1.308
Resistance of dewormers/fly control	3.68	1.069
A lack of experts trained in economics, production practices, and science	3.68	1.108
Value add solutions (e.g., distributors)	3.68	1.145
Lack of interest from general public	3.64	1.150
Facilities management	3.64	0.810
Global economy	3.64	0.757
Transportation	3.60	1.000
Knowing when to sell, expand, or hold steady	3.60	1.080
Animal rights	3.60	1.258
Breed selection for situation	3.60	0.957
Agriculture contributions to environmental issues (e.g., climate change, greenhouse gases, water quality, air quality, etc.)	3.56	1.158
Production capacity	3.56	0.821
Market fluctuations (lack of stability)	3.52	0.918
Use of antimicrobial substances	3.52	0.872

Using the 91 remaining items as a foundation, during round three panelists were given the opportunity to generate consensus around the most critical animal and food issues. As a result, consensus ranged from 50% to 100%, with 35 issues falling below the 80% cutoff point. As shown in Table 2, the remaining 56 issues that were retained created the final list of critical issues. Notably, nine critical issues received 100% consensus. Eight retained issues received 95% consensus, eight others received 90% to 94%, nine received 85% to 89%, and 22 received 80% to 84%. Although concern about the environment rose in importance during the last round, critical issues in the final list continued to lean heavily toward the next generation of the industry's workforce and the industry's image. Issues such as market fluctuations, political influences, and trade and price changes were considered least critical at the present moment.

**Table 2.** Delphi round three results: level of consensus for critical animal and food issues (*n* = 56)

Issue	Consensus %
Young people returning to food animal operations	100.00%
Telling our story as an industry	100.00%
Recruiting the next generation of agricultural leadership with a work ethic	100.00%
Qualified graduates	100.00%
Public perception of food animal industry	100.00%
Labor availability	100.00%
Environmental loads and sustainability (e.g., protection, stewardship, etc.)	100.00%
Consumer perception of food animal industry	100.00%
Consumer acceptance of current and future production practices (e.g., food safety, animal welfare, antibiotics, genetics, etc.)	100.00%
Long term economic prospects for animal agriculture (economic sustainability)	95.00%
Relating to the next generation of consumers	95.00%
Personnel wanting to work in production agriculture.	95.00%
Government regulations	95.00%
Disconnect between production agriculture and general public	95.00%
Animal welfare	95.00%
Misinformation on animal production practices	95.00%
Industry image	95.00%
Workforce preparation	94.74%
Training a sufficient number of food animal production students to meet the future needs of the industry.	94.74%
Awareness of animal protein's role in a healthy diet	90.00%
Need intern program set up for production agriculture	90.00%
Education of consumers on where food comes from	90.00%
Different perception between producers and consumers	90.00%
Anti-animal agriculture activism	90.00%
Animal well being	90.00%
Misinformed Consumers	89.47%
Public opinion about meat consumption	85.00%
Profitability of agriculture at the producer level	85.00%
Negative perception of animal agriculture	85.00%
Low profit margin	85.00%
Insufficient processing capacity	85.00%
Need for improved traceability and record keeping	85.00%
Food safety (e.g., safety of meat products)	85.00%
A lack of experts trained in economics, production practices, and science	85.00%
Value add solutions (e.g., distributors)	84.21%
Use of antimicrobial substances	84.21%
Societal demographic shift toward urban areas and the decrease in population and political clout in rural agricultural areas	84.21%
Reproductive efficiency	84.21%
Fear based information distributed to consumers	84.21%
Public relations	83.33%
Public perception of animal impact on climate change	80.00%
Interactions between diet and human well-being/health (mediated by microbiome)	80.00%
Health and robustness of farm animals	80.00%
Global economy	80.00%
Capital intensive (rising input costs)	80.00%
Product quality	80.00%
Poor legislative influence	80.00%
Poor consumer understanding of agriculture	80.00%
Misinformation on internet	80.00%
Marketing (of food production generally)	80.00%
Food security	80.00%
Feed efficiency	80.00%
Continued increasing demand for improved efficiency	80.00%
Agriculture contributions to environmental issues (e.g., climate change, greenhouse gases, water quality, air quality, etc.)	80.00%
Adapting to new technologies	80.00%
Gap between academia and industry	80.00%

Using the 56 remaining critical issues retained in round three, the CCM fostered the emergence of meaning and higher-order themes (Glaser, 1965; Glaser and Strauss, 1967). The critical issues were compared and preliminary codes such as “next generation,” “public perception,” and “political issues” quickly morphed into categories that shared common themes. The original categories were as follows: 1) Industry Image and Relationship with the Public (21 issues), 2) Animal and Human Health/Well-being (7 issues), 3) Production and Distribution Efficiency (7 issues), 4) Economic Concerns (6 issues), 5) Workforce Development and Personnel (5 issues), 6) Next Generation Pipeline (4 issues), 7) Government Regulations and Relationship with Legislative Leaders (3 issues), 8) Environmental Concerns (2 issues), and 9) Relationship with Higher Education/Researchers (1 issue). The constant comparison of codes and categories yielded similarities and distinctions that caused the merging of two groups. The “Workforce Development and Personnel” and “Next Generation Pipeline” categories were combined into one “Workforce Development and Pipeline” category as some issues, such as “workforce prep,” related to both categories and could therefore be coded either way. Likewise, “Economic Concerns” and “Environmental Concerns” were combined into one category named “Economic and Environmental Sustainability,” reducing the final list from nine to seven thematic categories as shown in Table 3.

**Table 3.** Results thematically categorized based on critical animal and food issues (*n* = 56).

Categories	Number of issues overall	Number of issues with 90–100% agreement
*Industry image and relationship with the public*	21	12
Telling our story as an industry		
Public perception of food animal industry		
Consumer perception of food animal industry		
Consumer acceptance of current and future production practices (e.g., food safety, animal welfare, antibiotics, genetics, etc.)		
Relating to the next generation of consumers		
Disconnect between production agriculture and general public		
Misinformation on animal production practices		
Industry image		
Awareness of animal protein's role in a healthy diet		
Education of consumers on where food comes from Different perception between producers and consumers		
Anti-animal agriculture activism		
Misinformed consumers		
Public opinion about meat consumption		
Negative perception of animal agriculture		
Fear based information distributed to consumers		
Public relations		
Public perception of animal impact on climate change		
Poor consumer understanding of agriculture		
Misinformation on internet		
Marketing (of food production generally)		
*Workforce development and pipeline*	9	8
Qualified graduates		
Labor availability		
Personnel wanting to work in production agriculture		
Workforce preparation		
A lack of experts trained in economics, production practices, and science		
Young people returning to food animal operations		
Recruiting the next generation of agricultural leadership with a work ethic		
Training a sufficient number of food animal production students to meet the future needs of the industry		
Need intern program set up for production agriculture		
*Economic and environmental sustainability*	8	2
Long term economic prospects for animal agriculture (economic sustainability)		
Profitability of agriculture at the producer level		
Low profit margin		
Global economy		
Capital intensive (rising input costs)		
Food security		
Environmental loads and sustainability (e.g., protection, stewardship, etc.)		
Agriculture contributions to environmental issues (e.g., climate change, greenhouse gases, water quality, air quality, etc.)		
*Animal and human health/well-being*	7	2
Animal welfare		
Animal well being		
Food safety (e.g., safety of meat products)		
Use of antimicrobial substances		
Interactions between diet and human well being/health (mediated by microbiome)		
Health and robustness of farm animals		
Product quality		
*Production and distribution efficiency*	7	0
Insufficient processing capacity		
Need for improved traceability and record keeping		
Value add solutions (e.g., distributors)		
Reproductive efficiency		
Feed efficiency		
Continued increasing demand for improved efficiency		
Adapting to new technologies		
*Government regulations and relationship with legislative leaders*	3	1
Government regulations		
Societal demographic shift toward urban areas and the decrease in population and political clout in rural agricultural areas		
Poor legislative influence		
*Relationship with higher education/Researchers*	1	0
Gap between academia and industry		

## DISCUSSION

Thematic analysis of the remaining critical issues and their corresponding categories provides insight into even further delineation of what animal and food experts deem important. Based on the data, deeper properties of the “Industry Image and Relationship with the Public” category relate to consumer knowledge about aspects of the industry, sources of that knowledge, and what consumers will accept and expect after receiving knowledge. There are differences between perceptions of industry representatives and those of consumers (e.g., Mahoney et al., 2020). Thus, how the industry presents itself, the information shared on its behalf, and its interactions with the public are critical as the industry finds itself in an educator role, and not just in that of production and distribution. Dimensions of the “Animal and Human Health/Well-being” category relate to the health and safety of animals and consumers due to their direct connection. In addition to the need to decrease risk of sickness and disease in both, there is also a need to decrease the liability of profit loss in any stage of production and distribution. The “Workforce Development and Pipeline” category points to both the desire and skillset of future industry employees who want to carry forward the success of the industry with responsibility, accurate knowledge, and a strong work ethic. Critical issues in the “Economic and Environmental Sustainability” category seem to highlight economic issues at local and global levels. The category also reveals that industry experts are also concerned about environmental impacts, not just consumers. The fact that producers feel pressure to be more efficient is evident in the “Production and Distribution Efficiency” category. Help is needed in this area in the form of advanced technology as well as enhanced feed resource management, reproduction, distribution, and tracking procedures. “Government Regulations and Relationship with Legislative Leaders” is a category underscoring current and future opportunities due to urbanization that affects political influence and regulations, which affect the industry as a whole and are sometimes impacted by consumer demand. Lastly, the “Relationship with Higher Education/Researchers” category brings attention to academia possibly being too far removed from industry to have proper knowledge of critical issues impacting industry decisions and influence with industry and political leaders.

While addressing all critical issues is needed and aspirational, a dichotomy exists between what issues generated the most agreement and those that were less agreed upon. This notable difference may serve as a beneficial guide for where experts believe preparation should begin to face industry challenges and opportunities between now and the year 2050. Based on data from the top 25 critical issues (i.e., critical issues with 90–100% agreement in round three of the Delphi method), “Industry Image and Relationship with the Public” (12 issues in the top 25) and “Workforce Development and Pipeline” (8 issues in the top 25) are vital starting points followed by “Economic and Environmental Sustainability” (2 issues in the top 25), “Animal and Human Health/Well-being” (2 issues in the top 25), and “Government Regulations and Relationship with Legislative Leaders” (1 issue in the top 25). With approximately 38% of representation in the overall list of critical issues and 48% in the list of top 25 critical issues, it is recommended that steps be made in relation to the “Industry Image and Relationship with the Public” category. Such steps relate to continued research involving where consumers receive their knowledge about the food animal industry, decisions about what stories the industry would like to tell and misconceptions it would like to dispel, and relevant methods to reach consumers with such information. Continued efforts to strengthen relationships with the public can overlap with other issues deemed critical. For example, communicating that the industry is also concerned about environmental, animal health, and consumer safety and well-being issues relate to both “Industry Image and Relationship with the Public” and “Animal and Human Health/Well-being” categories. Additionally, as an outreach component of the land-grant university system, Extension efforts can not only continue helping with public interaction, but also any lingering gaps relating to the industry's relationship with higher education. Enhanced collaboration between the industry and higher education will also be valuable to tackle the need for an available and well-trained workforce. Promoting the industry as a viable employment option to today's young people, recruiting a diverse pool of potential employees, and re-thinking what knowledge can be transferred in online or distance learning environments and what is essential for in-person training, are but a few suggestions for the field to continue considering now and in time to come. Lastly, research methods, such as the Delphi technique, that combine the insight of quantitative and qualitative methodologies, offer opportunities for future research to complement hard-science findings and corresponding publications.

## CONCLUSION

The study at hand explored today's most critical issues facing the animal and food industry. Using Delphi and CCMs, issues were identified and analyzed that could not only assist the industry in remaining strong now, but that could ensure its longevity in the next 30 years and beyond. This type of research empirically confirms the type of challenges and opportunities on the horizon and aids in the process of building more necessary bridges between theory and practice.
